# Successful Percutaneous Closure of a Large Saphenous Vein Graft Aneurysm With Vascular Plug 4, Optimal Percutaneous Device Selection

**DOI:** 10.7759/cureus.14016

**Published:** 2021-03-21

**Authors:** Kazuyuki Ishibashi, Mamika Motokawa, Noboru Motomura

**Affiliations:** 1 Cardiovascular Surgery, Toho University Sakura Medical Center, Sakura, JPN; 2 Cardiovascular Surgery, Goi Hospital, Ichihara, JPN

**Keywords:** aneurysm, vein graft, coronary artery bypass grafting(cabg)

## Abstract

Saphenous vein graft (SVG) aneurysm is one of the late unusual complications of coronary artery bypass grafting (CABG). We report a case of a very large SVG aneurysm successfully treated with a vascular plug 4. A 94-year-old man was referred to our hospital for an expanding aortic aneurysm. The patient had undergone CABG 24 years prior, with three SVGs to the left anterior descending (LAD) artery, left circumflex arteries (LCX) artery, and right coronary artery (RCA). A computed tomography scan of the chest demonstrated an 8.8 cm × 6.5 cm aneurysm arising from an ascending aortic wall. Coronary angiography revealed an aneurysm arising from the proximal segment of the SVG to the RCA. The distal graft anastomosis of the SVG to the RCA and LCX was occluded. Another SVG to the LAD was widely patent. From a radial approach, a 5-Fr catheter (Tempo, Cardinal Health, OH) was advanced into its cavity, and a 7-mm Amplatzer vascular plug 4 (AVP-IV, AGA Medical Corporation, MN) was successfully delivered. Contrast CT confirmed a thrombus development in the cavity. The patient was discharged home. However, interventions utilizing vascular plug insertion are limited to cases of graft occlusion. We report a case of the successful treatment of a large SVG aneurysm using the Amplatzer vascular plug 4.

## Introduction

Despite the advances in percutaneous coronary intervention (PCI) technology, drug therapy, and primary preventive strategies, including lifestyle changes, coronary artery bypass graft (CABG) are still one of the most common treatments for ischemic heart disease globally [1]. CABG typically uses the internal thoracic artery (ITA) to the left anterior descending (LAD) branch. However, in other areas, although the 10-year patency rate is inferior to that of ITA due to various reasons, saphenous vein grafts (SVGs) are still considered valuable graft materials and are used in many cases worldwide [2]. It is known that the use of vein grafts rarely leads to the development of aneurysms in the acute and late phases.

Aneurysms that develop in the acute postoperative period are mostly due to technical factors related to surgical procedures, such as defects in the harvest. On the other hand, the late onset of aneurysms is mainly due to age-related degeneration of the vascular wall [3]. Traditionally, SVG aneurysms are treated surgically [4,5]. However, due to the need for reoperations and patients' advanced age, various new treatments have been reported in recent years [3,6].

We present a case of SVG aneurysm near the aortic anastomosis, which was detected ≥20 years after a CABG and was successfully thrombosed percutaneously using an Amplatzer vascular plug 4 (AVP4).

This article was previously presented as a meeting abstract and poster at the 18th Congress of Asian Society for Vascular Surgery and the 12th Asian Venous Forum.

## Case presentation

**Figure 1:**
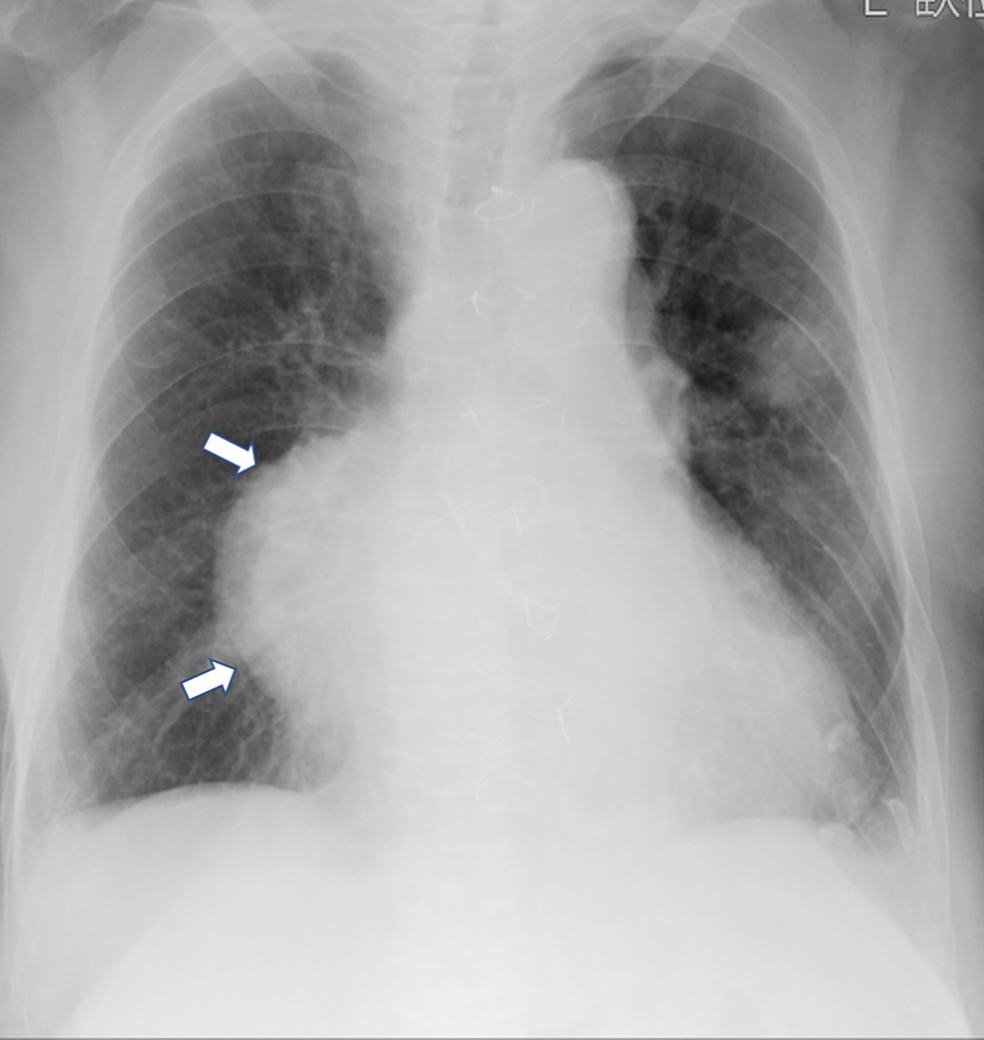
Chest X-ray findings at admission. The chest X-ray shows a large mediastinum and right pericardiac mass (white arrow).

A 94-year-old man was referred to our hospital due to the presence of an abnormal shadow in the chest X-ray (Figure 1). He had no chest symptoms and was admitted to our hospital.

**Figure 2:**
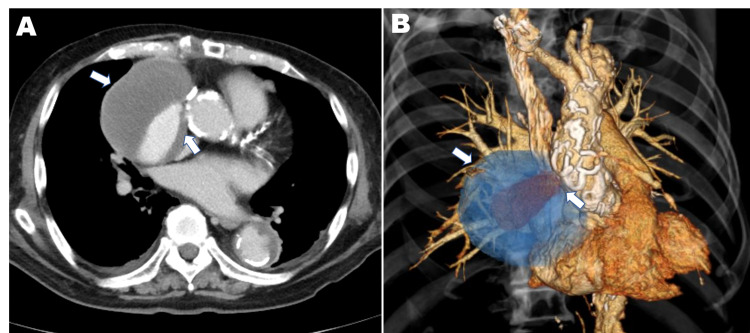
CT scan findings. (A) The CT scan shows a saphenous vein aneurysm filled with thrombus within a patent cavity communicating with the ascending aorta. (B) Three-dimensional construction CT shows the relationship of the aneurysm cavity to the ascending aorta.

The patient had undergone CABG 24 years ago, with an SVG each to the LAD, left circumflex (LCX), and right coronary (RCA) arteries. A chest X-ray revealed a right mediastinal mass which grew in size over the years. A CT scan of the chest revealed a partially thrombosed aneurysm (8.8 cm × 6.5 cm) arising from the site of the SVG near the ascending aortic wall (Figures 2A, 2B). Coronary angiography revealed an aneurysm from the SVG proximal segment to the RCA that did not communicate with the distal RCA. The SVG anastomosis to the LAD was widely patent, and that to the LCX was completely occluded. Given the patient's advanced age, risks associated with a repeat cardiac surgery, and absence of angina, we opted to close the aneurysm with a vascular plug.

**Figure 3:**
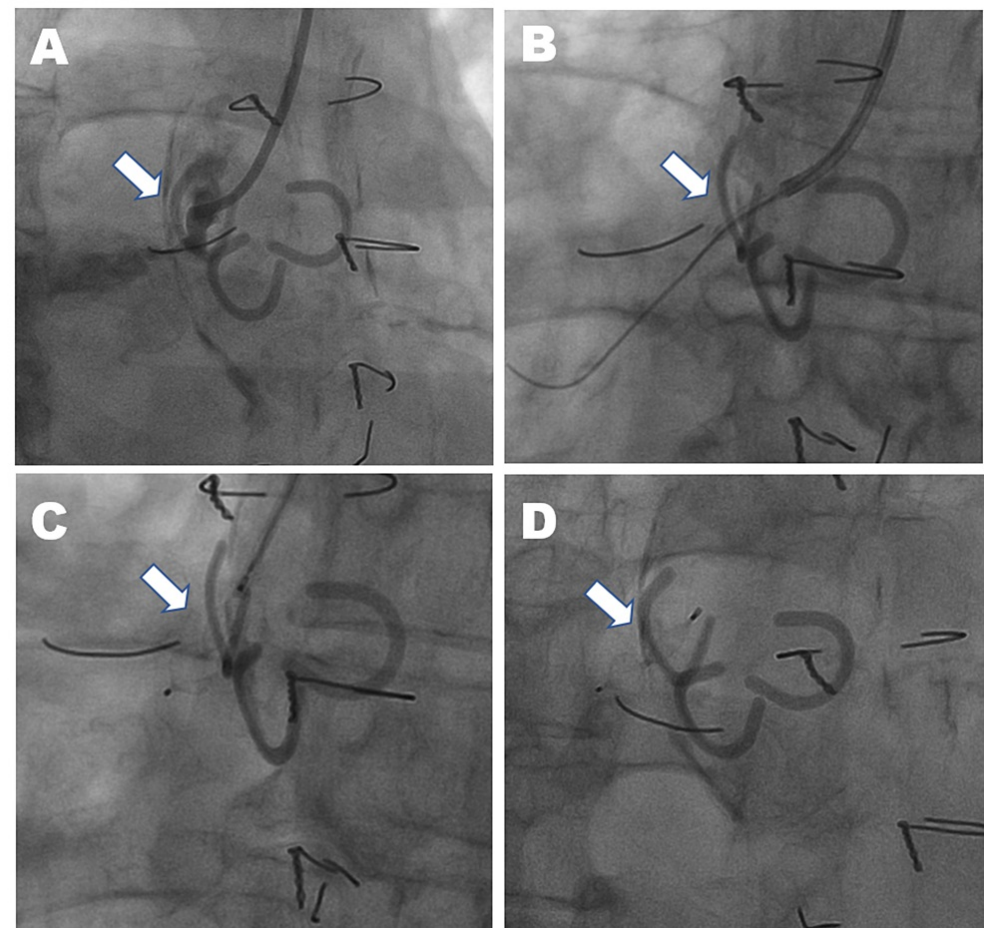
Angiography of the RCA performed from a right radial approach. (A) A short neck and a large graft aneurysm can be seen in the SVG to the RCA. (B) A stiff wire is inserted into the aneurysm through the sheath. (C) A vascular plug is extruded through a 5 Fr catheter in the sheath. (D) The vascular plug successfully occludes the SVG to the RCA. RCA: Right coronary artery; SVG: saphenous vein graft

From the right radial approach, a 0.035-inch" guidewire was inserted into the SVG aneurysm. A 6-Fr guiding catheter (Terumo Medical Corporation, Tokyo, Japan) was used to engage the SVG's neck, and coronary angiography was performed (Figure 3A).

A 5-Fr catheter (Tempo, Cardinal Health, OH) was advanced into its cavity. Next, the wires were removed, and a curved stiff guidewire (Cordis, Cardinal Health, OH) was inserted to provide maximal distal support (Figure 3B). The Amplatzer vascular plug 4 (AVP-IV, AGA Medical Corporation, MN) was inserted such that its proximal disk lay within the SVG and the distal disk within the aneurysmal neck (Figure 3C). Finally, a 7-mm AVP-4 was successfully deployed (Figure 3D).

**Figure 4:**
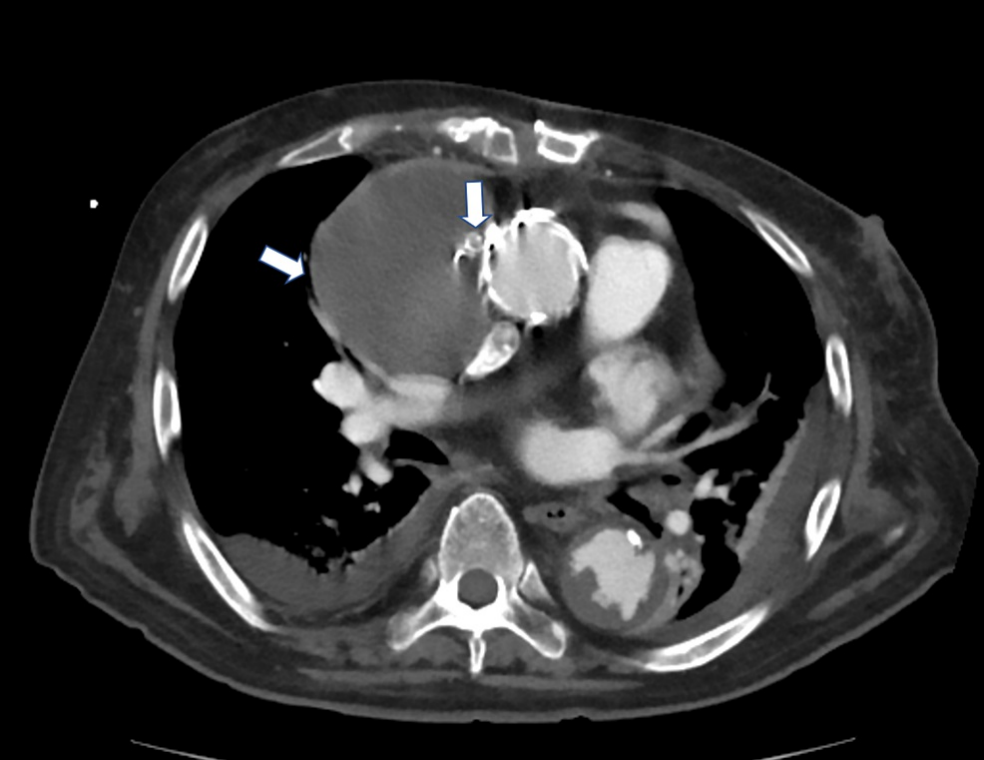
Postoperative CT. The inside of the aneurysm is completely thrombosed (white arrow).

A contrast CT was performed seven days later, which confirmed the presence of thrombus in the cavity (Figure 4).

The patient was discharged home. At the six-month follow-up, recovery courses were uneventful, and the patient had no symptoms. However, one year after the procedure, he died of old age.

## Discussion

An SVG aneurysm is a very rare complication of CABG surgery occurring in 0.07% of patients [7]. Patients with SVG aneurysms are often asymptomatic, and the aneurysms are accidentally identified by chest X-ray, as in this case. Therefore, the actual number of cases may be higher than what is reported. Because of the possibility of sudden death when the patient presents with chest pain, shortness of breath, hemoptysis, or heart failure due to fistula in the right heart system, SVG aneurysm is one of the rare and serious complications that must always be considered after CABG surgery in the late phase. When an SVG aneurysm is suspected, diagnostic imaging such as CT, magnetic resonance imaging, and selective coronary angiography is extremely important. Although there are no clear criteria for surgical treatment, it is generally considered when the mass diameter is >2 cm or when the patient presents with symptoms of rupture. One report describes an increase in the diameter of an aneurysm from 2 cm to 8 cm in four years [3]. Although surgical repair with re-sternotomy is a reliable procedure, it is a reoperation, and the patient may not be able to tolerate the surgery for various reasons, including old age. With newly developed devices, various percutaneous treatments for SVG aneurysms have been reported in recent years [6].

However, a key aspect of these treatments is selecting the right device based on the location and morphology of the aneurysm. Another major factor is the need for revascularization from the SVG aneurysm to the distal coronary artery. If a distal bypass is required and there is sufficient proximal and distal landing zone, a covered coronary stent is an optimal option [6,8,9]. Coil embolization is also indicated for small saccular aneurysms and when the distance from the aortic wall of the SVG to the aneurysm is long, but it cannot be used for short necks or huge aneurysms [6,10]. A method replacing the aortic stent graft in the ascending aorta to cover the SVG origin has also been reported [11]. However, using it in our case was difficult, since the SVG proximal site in the graft to the LAD was extremely close to the SVG aneurysm's origin and could have occluded the patent graft. If the aneurysm was close to the ascending aorta, an occluder device for patent ductus arteriosus or ventricular septal defect could have been considered [12,13]. However, using an occluder device was difficult because the proximal site of SVG anastomosis orifice was approximately 10 mm based on the coronary angiography. Therefore, since revascularization to the distal coronary artery is unnecessary, a vascular plug has been used to occlude the origin of SVG to thrombus the aneurysm [14,15].

Since the Amplatzer vascular plug 2 (St. Jude Medical, St. Paul, MN) required a 7-Fr guiding catheter (Terumo Medical Corporation, Tokyo, Japan) or longer and could potentially protrude into the aorta when released, we used plug 4, which could be released using a shorter 5-Fr catheter it also had a softer device. Compared to plug 2, plug 4 is thinner and more flexible, making it easier to be delivered into the flexed vessels with a 5-Fr catheter. If the catheter can be advanced to the proximal neck, it becomes easy to place the AVP 4. However, since the maximum diameter of the AVP 4 is 8 mm, it can be used only for blood vessels ≤ 6 mm in diameter. For blood vessels >-6 mm, it is necessary to use the AVP 2. In particular, it is technically difficult to insert a catheter when the entrance is highly flexed. If the device is forced forward, there is a possibility of vascular damage; thus, care must be taken when selecting the right device. Several case reports discussing various modes of treatment of SVG aneurysms are available in the literature. However, in the absence of proper guidelines, it is essential that the heart team, including cardiologists, cardiovascular surgeons, and radiologists, discuss the timing of the intervention.

## Conclusions

SVG aneurysm is a rare but typical late complication after CABG surgery, with the potential for high morbidity and mortality. Adequate preoperative imaging evaluation, accounting for mechanical complications, aneurysm morphology, and coronary artery hemodynamic distribution, is critical when considering treatment options. We have described a case of SVG aneurysm caused by aortic anastomosis, detected ≥ 20 years after a CABG operation. We were able to form a thrombus formation at the aneurysm site using the AVP 4. When treating an SVG aneurysm, it is important to choose the optimal method, percutaneous or surgical, based on the patient's medical history and clinical pathology.
